# Molecular approaches for manipulating astrocytic signaling *in vivo*

**DOI:** 10.3389/fncel.2015.00144

**Published:** 2015-04-21

**Authors:** Alison X. Xie, Jeremy Petravicz, Ken D. McCarthy

**Affiliations:** ^1^Department of Pharmacology, School of Medicine, University of North Carolina at Chapel HillChapel Hill, NC, USA; ^2^Department of Brain and Cognitive Sciences, Picower Institute for Learning and Memory, Massachusetts Institute of TechnologyCambridge, MA, USA

**Keywords:** astrocyte, *in vivo*, GPCR signaling, DREADD, IP3R2 KO

## Abstract

Astrocytes are the predominant glial type in the central nervous system and play important roles in assisting neuronal function and network activity. Astrocytes exhibit complex signaling systems that are essential for their normal function and the homeostasis of the neural network. Altered signaling in astrocytes is closely associated with neurological and psychiatric diseases, suggesting tremendous therapeutic potential of these cells. To further understand astrocyte function in health and disease, it is important to study astrocytic signaling *in vivo*. In this review, we discuss molecular tools that enable the selective manipulation of astrocytic signaling, including the tools to selectively activate and inactivate astrocyte signaling *in vivo*. Lastly, we highlight a few tools in development that present strong potential for advancing our understanding of the role of astrocytes in physiology, behavior, and pathology.

## Introduction

G protein coupled receptors (GPCRs) are the primary molecules through which non-excitable cells transduce information from external cues to biological responses. There are four major families of GPCRs that are distinguished by their composition, ability to activate intracellular signaling cascades, and the functional consequences associated with their activation. The importance of GPCRs is underscored by the fact that these molecules are the most common targeted class of proteins of therapeutic agents. Astrocytes express each of the major classes of GPCRs (Porter and McCarthy, 1997) clearly demonstrating that these cells are dynamically coupled to the activity of their surrounding cellular and chemical milieu. It is likely that astrocyte GPCRs are activated by neurotransmitters released from neurons synaptically as well as through volume transmission. It is also likely that neighboring non-neuronal cells including microglia, vascular endothelial cells, astrocytes, and other resident CNS cells release molecules that activate astrocyte GPCRs and modulate astrocyte activity. In certain situations, low levels of ambient neurotransmitters might tonically activate astrocyte GPCRs. However, it is likely that in most cases astrocyte GPCRs are spatially restricted to discrete signaling domains that are activated with different temporal characteristics dependent on the source of the signal and biological response being affected. Layered onto this signaling complexity is the morphological complexity of astrocytes; the fine processes of an individual astrocyte within the CA1 region of the hippocampus can associate with ~100,000 synapses (Bushong et al., 2002). This being the case, different regions of an individual astrocyte are likely responding simultaneously to local signals (from neurons or other cell types) with different functional outcomes. Collectively, this information underscores how difficult it is to replicate *in vitro* or *in situ* the complicated pattern of GPCR activation that normally occurs *in vivo*.

**Figure 1:**
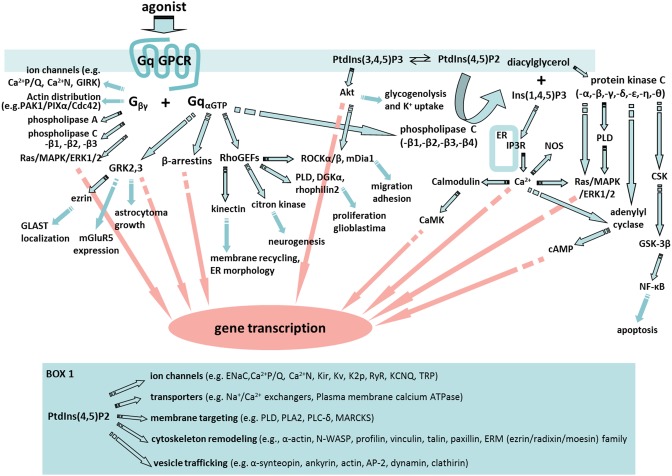
**A fabric of signaling cascades can be activated by astrocyte Gq-GPCRs**. Upon Gq-GPCR activation, Gαq subunits are known to interact with G protein-coupled receptor kinases (GRKs), β-arrestins, Rho family of guanine exchange factors (RhoGEFs) and phospholipase C (PLC) in astrocytes or astroglia. In addition, Gβγ subunits are able to regulate ion channel properties, as well as to interact with signaling molecules including cdc42, PAK-PIXα, phospholipase A (PLA), PLC, and Ras/MAPK/ERK1/2. Many of the key signaling molecules regulated by Gβγ are expressed in astrocytes or astroglia. Therefore, Gq-GPCR activation in astrocytes is likely to activate an entire fabric of downstream signaling pathways that may include (1) GRKs-mediated glutamate transporter (GLAST) localization (Nijboer et al., 2013), gene transcription (Atkinson et al., 2009), metabotropic glutamate receptor 5 (mGluR5) expression (Sorensen and Conn, 2003), and astrocytoma growth (Woerner et al., 2012); (2) β-arrestin-mediated gene silencing (McLennan et al., 2008; Miyatake et al., 2009; Zhu and Reiser, 2014); (3) RhoGEFs-mediated activation of Kinectin (Santama et al., 2004), citron kinase (Ackman et al., 2007), phospholipase D (PLD) (Burkhardt et al., 2014), diacylglycerol kinase (DGKa) (Kefas et al., 2013), Rhophilin2 (Vedrenne and Hauri, 2006; Danussi et al., 2013), Rock (Holtje et al., 2005; Lau et al., 2011), and mDia1 (Shinohara et al., 2012); (4) PLC regulation of PtdIns(3,4,5)P3 (PIP3)/protein kinase B (PKB or Akt) pathway (DiNuzzo et al., 2013; Kong et al., 2013), PtdIns(4,5)P2(PIP2)/diacylglycerol (DAG)/protein kinase C (PKC) pathway, and PIP2/Ins(1,4,5)P3(IP3) pathway. Moreover, PKC activation in astrocytes (Wang et al., 2002) engages PLD (Servitja et al., 2003), c-src tyrosine kinase (CSK) (Jo et al., 2014), glycogen synthase kinase (GSK) (Sanchez et al., 2003), and cAMP signaling. Many of these signaling pathways are known to trigger cellular responses that are important for astrocyte function including gene transcription and cell migration. Box 1. Selected PIP2-induced signaling pathways in which key molecules are expressed by astrocytes. These signaling molecules include: Epithelial sodium channel (ENaC) (Miller and Loewy, 2013), PLA2 (Ha et al., 2014), Myristoylated alanine-rich C-kinase substrate (MARCKS) (Vitkovic et al., 2005), Wiskott–Aldrich syndrome protein (WASP) (Murk et al., 2013), profilin (Molotkov et al., 2013), vinculin, talin, and paxillin (Kalman and Szabo, 2001), ERM protein family (Persson et al., 2010), α1-syntrophin (Masaki et al., 2010), ankyrin (Lee et al., 2012), adipocyte protein 2 (AP2) (Rossello et al., 2012), and clathirin (Pascual-Lucas et al., 2014).

By far, the emphasis in astrocyte GPCR signaling activity has been on the regulation of Ca^2+^. This is not surprising given that Ca^2+^ fluxes play a very important role in regulating biological processes and Ca^2+^ is the only signaling molecule that we can readily monitor selectively in astrocytes in complex tissue such as brain. Consequently, we know a lot about astrocyte Ca^2+^ responses following the activation of Gq-GPCRs and the consequences of increasing astrocyte Ca^2+^ by a number of different approaches. Two important points need to be kept in mind when considering findings in this area. First, most investigations linking increases in astrocyte Ca^2+^ with functional responses use pharmacological methods to increase astrocyte Ca^2+^ and consequently findings may not reflect physiological responses. Second, while the field has focused on the role of astrocyte Ca^2+^ in functional responses, the activation of Gq-coupled GPCRs leads to the modulation of a broad set of signaling cascades beyond changes in Ca^2+^; the variety of effector proteins in Gαq “interactome” may affect astrocyte Ca^2+^ responses as well as play important roles in physiological responses to Gq-GPCR activation (Figure 1; Sanchez-Fernandez et al., 2014).

## Molecular tools for selective activation of astrocytes *in vivo*

### Temporal control of astrocyte activation *in vivo* using optogenetics

Optogenetics is an extremely powerful tool for activating and inactivating neuronal circuits *in vivo* with high temporal resolution. Optogenetic regulation of neuronal activity generally occurs through the flux of ions that either depolarize or hyperpolarize neurons with high temporal and spatial resolution (Schoenenberger et al., 2011; Lin, 2012). More recently, a number of investigators have used this technology to activate astrocyte signaling *in situ* and *in vivo* (Gourine et al., 2010; Perea et al., 2014; Yamashita et al., 2014). It is important to understand that while optogenetic activation of astrocytes can lead to changes in ion fluxes across plasma and intracellular compartment membranes, these fluxes do not remotely reflect the changes in signaling that occur following the activation of astrocyte GPCRs, the primary mode for activating astrocyte signaling (Agulhon et al., 2012; Sanchez-Fernandez et al., 2014).

Multiple variants of channelrhodopsin (ChR2) have been expressed in astrocytes, primarily to elicit Ca^2+^ responses (Table 1). *In vivo*, activation of the ChR2 variant ChR2 (H134R) (Nagel et al., 2005) in astrocytes in the ventral surface of the medulla oblongata was shown to lead to increases in intracellular Ca^2+^ and alteration in respiratory activity of rats (Gourine et al., 2010). This same variant of ChR2 was employed to illustrate the ability of astrocyte Ca^2+^ elevations to alter the firing and orientation responses of mouse primary visual cortex excitatory and inhibitory neurons *in vivo* (Perea et al., 2014). Most recently, Beppu et al. (2014) created mice expressing the ChR variant ChR2 (C128S) or an optogenetic proton pump (ArchT). Activation of these optogenetic tools lead to, respectively, acidification or alkalization of astrocytes which modulated glutamate release and ischemic damage *in vivo*. The remainder of studies using ChR2 or its variants have been performed using culture or slice preparations, but provide valuable insights into the mechanisms of their ability to activate astrocytes signaling (Figueiredo et al., 2014). A second variant, CatCh (Ca^2+^ translocating ChR, which has improved Ca^2+^ permeability) (Kleinlogel et al., 2011) when expressed in cultured astrocytes, was found to also increase Ca^2+^ but with varying reliability compared to ChR2 (H134R) (Li et al., 2012; Figueiredo et al., 2014). A non-ChR2 light activated channel, the light-gated Ca^2+^-permeable ionotropic GluR6 glutamate receptor (LiGluR), has also been used in cultured astrocytes, with again varying results (Li et al., 2012).

**Table 1:**
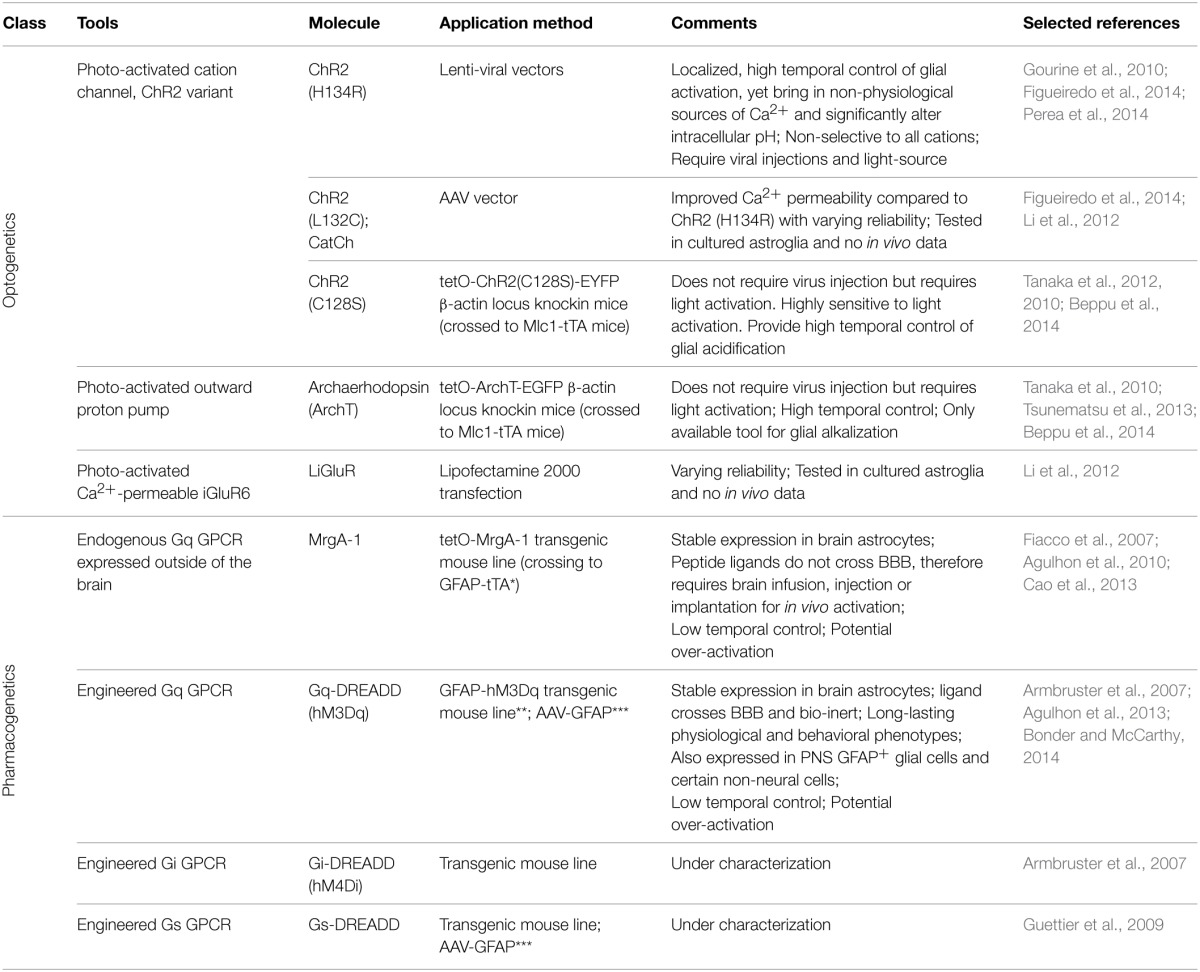
**Tools for selectively activation of astrocyte signaling *in vivo***.

^*^Mouse line available in the Jackson Laboratory, stock No. 005964; ^**^For various DREADD constructs and transgenic mouse lines currently available, see http://pdspit3.mml.unc.edu/projects/dreadd/wiki/WikiStart, ^***^AAV-GFAP minimal promotor available via UNC Vector core.

The advantages of using optogenetics to activate astrocytes are the ability to temporally and spatially control the extent of activation. However, there are a number of important caveats involved in the use of optogenetics to activate astrocyte signaling cascades. First, it is unresolved as to the source of Ca^2+^ when using these optogenetic actuators in astrocytes. Extracellular Ca^2+^ entry through ChR2 variants seems the most logical mechanism, but a recent comparative study found that intracellular stores are the primary source (Figueiredo et al., 2014). These finding are at odds with studies from other laboratories (Li et al., 2012), and previous research from the same authors (Gourine et al., 2010). Current evidence indicates that both external and internal Ca^2+^ sources are involved in the ChR2 induced Ca^2+^ signal. Second, ChR2 and its variants are non-selective cation channels, allowing the entry of Na^+^ K^+^, H^+^, and Ca^2+^ upon activation (Nagel et al., 2003). ChR2 expressed in the cultured astroglia cell line GL261 display significant influx of Na^+^ and Ca^2+^ across the cell membrane based upon fluorescent indicator imaging (Ono et al., 2014). Additionally, significant alterations in intracellular pH were also observed in the same study, which has been corroborated in the recent study by Beppu et al. (2014). Interestingly, the ChR2 (H134R) used in the two current *in vivo* studies of astrocyte function was engineered to increase its ability to depolarize cultured HEK293 and neuronal cells over wild type ChR2 (Nagel et al., 2005; Beppu et al., 2014). Astrocytes process intracellular changes in Na^+^, Ca^2+^, and protons in different ways that may affect several different astrocyte functions. For example, increases in intracellular sodium and membrane depolarization, which is known to occur with ChR2-stimulation (Gourine et al., 2010), could alter glutamate reuptake efficacy (Djukic et al., 2007; Unichenko et al., 2012; Verkhratsky et al., 2013). Alterations to intracellular pH in astrocytes are important in the regulation of ischemic damage *in vivo* (Benesova et al., 2009; Beppu et al., 2014) and gap junction connectivity (Duffy et al., 2004). It is not unreasonable to hypothesize that ChR2 activation leads to membrane depolarization of astrocytes and interferes with a number of transport systems, most importantly glutamate, which may lead to effects on neuronal activity. In addition, to use optogenetics *in vivo* it is necessary to use a viral vector to deliver the optogenetic construct and may require the insertion of a light probe to activate the optogenetic channel in deeper brain regions. It seems very likely that these insults will lead to pathological changes that alter astrocyte responses. Overall, while optogenetic tools are great tools to activate or silence neurons, using optogenetics to increase astrocyte Ca^2+^ bypasses the majority of signaling pathways that are activated by GPCRs, as well as result in aberrant ion fluxes that likely never occur under physiological conditions (Fiacco et al., 2007; Agulhon et al., 2012; Wang et al., 2013).

### Selective activation of astrocyte Gq-GPCR signaling with MrgA-1

The first experimental system that enabled the specific activation of endogenous astrocyte signaling cascades in complex tissue was the Mas-related gene A1 (MrgA-1) transgenic mouse line (Fiacco et al., 2007) (Table 1). MrgA-1 is a Gq-GPCR that is normally expressed in a subsets of nociceptive sensory neurons (Dong et al., 2001) but not in the brain. The ligand used to activate MrgA-1, the peptide FMRF, does not activate endogenous brain Gq-GPCRs (Fiacco et al., 2007). To achieve cell specificity in the brain, MrgA-1 expression was controlled by using a tetracycline-controlled inducible expression system (tetO system), which requires a second transgene, the transcription transactivator (tTA). By crossing tetO-MrgA-1 transgenic mouse to GFAP-tTA transgenic mouse, GFAP^+^ glia become the only cells in the brain that express both tTA and tetO-MrgA-1, therefore the only CNS cells to express MrgA-1 receptor (Fiacco et al., 2007). As a native GPCR, MrgA-1 activation triggers the entire fabric of signaling cascades normally activated by endogenous astrocyte Gq-GPCRs; an important component that is absent when selectively increasing specific signaling molecules such as IP3 and Ca^2+^. With respect to Ca^2+^, MrgA-1 activation leads to a similar spatial and temporal response as endogenous Gq-GPCRs in hippocampal astrocytes (Fiacco et al., 2007).

MrgA-1 transgenic mice were used to prepare brain slices to test the gliotransmission hypothesis at hippocampal CA3-CA1 synapses (Fiacco et al., 2007; Agulhon et al., 2010; Wang et al., 2012, 2013; Devaraju et al., 2013). Initial reports showed that bath application of FMRFa induced widespread Ca^2+^ elevations in stratum radiatum astrocytes from MrgA-1 mice, while CA1 neuronal Ca^2+^ activity, excitatory synaptic transmission and short- or long-term excitatory synaptic plasticity in CA3-CA1 synapses were not affected (Fiacco et al., 2007; Agulhon et al., 2010). Later Wang et al. reported that although MrgA-1 mediated astrocyte activation did not change neuronal excitability and miniature excitatory synaptic currents (mEPSCs) in neurons near the surface of hippocampal slices, both bath and microinjection of FMRFa led to a transient hyperpolarization and decreased mEPSC frequency in neurons below 80 μm depth in the slices (Wang et al., 2012, 2013). These studies suggested that selective activation of Gq-GPCR signaling in astrocytes increased activity of the Na^+^/K^+^ ATPase, resulting in a reduction of extracellular K^+^ which consequently hyperpolarized neurons and suppressed excitatory transmission (Wang et al., 2012). The Gq-GPCR activated change in [K^+^] was hard to detect in the superficial layer of the slices, where the constant bath perfusion buffered the effects (Wang et al., 2012). The K^+^ removal hypothesis was supported by an independent study from Devaraju et al. who found that both Schaffer Collaterals stimulation and selective stimulation of astrocytic MrgA-1s potentiated inward K^+^ current and glutamate uptake in hippocampal astrocytes (Devaraju et al., 2013). These data suggest that astrocytic Gq-GPCR activation may regulate neuronal excitability and modulate neuronal network activity indirectly rather than inducing the release of gliotransmitters.

The MrgA-1 mouse line is rarely used for studying astrocyte function *in vivo* because the peptide agonists do not effectively cross blood brain barrier. Recently, Cao et al. used MrgA-1 mouse line to study the role of astrocytic activation in behavior by implanting infusion cannula or osmotic pumps into the brain of MrgA-1 mouse (Cao et al., 2013). Brain infusion of the peptide agonist of MrgA-1 mice induced antidepressant-like effect in forced swimming test and reversed depression-like behavior in MrgA-1 mice suggesting that astrocytic Gq-GPCR signaling is capable of modulating depressive-like behaviors (Cao et al., 2013).

### Pharmacogenetic activation of astrocytic signaling *in vivo* using DREADDs

In 2007, a new family of engineered GPCRs, Designer Receptor Exclusively Activated by Designer Drugs (DREADD) were developed (Armbruster et al., 2007) and have become the best option for activating GPCR signaling in specific cell populations *in vivo* (Rogan and Roth, 2011) (Table 1). A significant advantage of DREADDs compared to MrgA-1 is that the ligand of DREADDs, clozapine N-oxide (CNO), crosses BBB (Bender et al., 1994), therefore enabling non-invasive manipulation of receptor activity via peripheral injections (e.g., intraperitoneal or intravenous injections) and even via drinking water (Jain et al., 2013). The M3 muscarinic cholinergic receptor (M_3_AChR) was engineered through directed molecular evolution (Dong et al., 2010) that led to a striking decrease the affinity of this receptor for its native agonist (acetylcholine) as well as to a large increase in affinity for CNO. In addition, DREADDs do not exhibit constitutive activity and CNO is pharmacologically inert in the absence of DREADDs (Armbruster et al., 2007; Nichols and Roth, 2009; Dong et al., 2010). Consequently, mice expressing DREADDs do not exhibit a phenotype in the absence of CNO and CNO does not lead to a phenotype in wild type mice (Alexander et al., 2009; Guettier et al., 2009; Agulhon et al., 2013). Since their development, DREADDs have been extensively used to chronically and acutely activate (Gq-DREADDs) and silence (Gi-DREADDs) specific subsets of neurons *in vivo* (Wess et al., 2013).

Gq-DREADD was introduced into astrocyte research studies with the development of GFAP-Gq-DREADD mice for specifically activating GFAP^+^ glial Gq-GPCR signaling *in vivo* (Agulhon et al., 2013). Gq-DREADD expression was regulated by the 2.2 Kb human GFAP promotor fragment; a hemagglutinin (HA) tag was added to the N-terminus of the Gq-DREADD for highly specific antibody staining. Extensive immunostaining studies demonstrated that the expression of Gq-DREADD was restricted to GFAP^+^ glia in the CNS and PNS (Agulhon et al., 2013). Bath application of CNO *in situ* or i.p. injection of CNO *in vivo* led to Ca^2+^ increases in brain astrocytes, without affecting Ca^2+^ in nearby neurons; CNO induced Ca^2+^ increases occurred throughout astrocytes including their fine processes within the neuropil. The development of this model enabled, for the first time, examination of the behavioral and physiological consequences of specifically activating Gq-GPCR signaling in GFAP^+^ glia. CNO administration to GFAP-Gq-DREADD transgenic mice revealed robust and unexpected behavioral and physiological phenotypes that were absent in litter mate controls; phenotypic changes include robust increases in heart rate and blood pressure, saliva formation, a decrease in body temperature, and increased sedation in the presence of a GABA receptor agonist (Agulhon et al., 2013). These findings suggest that GFAP^+^ glia have the potential for modulating a number of important physiological processes.

In addition to GFAP-Gq-DREADD mice, transgenic mouse lines expressing Gs- and Gi-DREADD specifically in GFAP^+^ glia were developed and are currently under characterization in the McCarthy laboratory. GFAP-DREADD transgenic mice offer the best system to non-invasively and simultaneously activate widely distributed astrocyte populations. Other systems for manipulating astrocytic activity *in vivo*, including MrgA-1 transgenic mice and optogenetics, requires direct application of ligand/light to brain tissue and thus have spatial limitations with regard to cells being activated at a given time. Region-specific expression DREADD can be achieved via viral delivery (Bull et al., 2014). Once the expression pattern is established, one can activate a subset of astrocytes acutely or chronically in dose-dependent manner, and behavior and physiological outcome can be measured from free-moving, awake mice. For the first time in astrocyte research, we can now test the contribution of astrocytes GPCR signaling in physiology and behavior, as well as verify the previously known astrocytic function in intact animals.

There are caveats associated with using pharmacogenetic systems to study the role of astrocyte Gq-GPCR signaling *in vivo*. First, engineered GPCRs are driven by an exogenous promoter system and consequently the levels of expression are likely to be different than that of endogenous astrocytic GPCRs. While this does not appear to lead to markedly different Ca^2+^ responses compared to endogenous receptors, it has not been confirmed that the engineered GPCR signaling cascades are regulated in a similar manner. Second, it is impossible to mimic the temporal and spatial characteristics of *in vivo* GPCR activation, a caveat associated with all pharmacological stimulation. Following an i.p. injection of CNO, most of the physiological phenotypes were observed in 5 min and peak in 30 ~ 45 min (Agulhon et al., 2013). Increased temporal and spatial resolution can be obtained by either microinjecting CNO into the region of interest or uncaging CNO with laser pulse activation; the latter approach has temporal and spatial resolution similar to optogenetic activation; caged CNO has recently been prepared (Brian Roth, personal communication). Overall, DREADD technology enables activation of the entire fabric of endogenous signaling cascades in specific cell types that are generally stimulated by GPCRs; this is a striking advantage over most other methods used to activate glial signaling *in situ* or *in vivo*.

### Selective astrocyte gene rescue in mice with global gene deletion

The optogenetic and pharmacogenetic approaches for activating astrocyte *in vivo* share a common pitfall of potential over-activation. Recently, a conditional endogenous gene repair approach was used to isolate the role of astrocyte-specific endogenous signaling *in vivo* in a mouse model of Rett's syndrome (RTT).

RTT is an X-chromosome-linked autism spectrum disorder due to the loss of function of the transcriptional regulator methyl-CpG-binding protein 2 (MeCP2) in the brain (Amir et al., 1999; Guy et al., 2007). Because MeCP2 is expressed in all CNS cell types (Ballas et al., 2009), a conditional knock-in mice, MeCP2^loxp^ mice, was developed to study cell-specific disease mechanisms. In this model, the endogenous *Mecp2* gene is silenced by insertion of a *lox*P*-Stop* cassette, but can be activated when combined with Cre- or Cre-ER system (Guy et al., 2007). When MeCP2^loxp^ mice were crossed to hGFAP-CreER^T2^ mice (Hirrlinger et al., 2006), the expression of MeCP2 was selectively restored in GFAP^+^ astrocytes when mice were treated with tamoxifen (Lioy et al., 2011). The specific re-expression of MeCP2 in astrocytes significantly improved RTT phenotype possibly by restoring normal dendritic morphology and levels of the excitatory glutamate transporter VGLUT1 (Lioy et al., 2011). This model illustrates the potential of using conditional astrocyte-specific rescue model to isolate the function of astrocyte signaling *in vivo* and in disease.

### Spatial control of astrocytic signaling via viral delivery—advantages and disadvantages

At this time, there are no astrocyte transcriptional units that can be used to target specific populations of astrocytes in mature brain. Consequently, a large number of investigators have used viral vectors to perturb signaling in subpopulations of astrocytes using both adeno-associated viruses (AAV) and lentiviral vectors (Figueiredo et al., 2014). Several AAV serotypes show tropism toward astrocytes, including AAV 2/5 and AAV 8 (Koerber et al., 2009; Aschauer et al., 2013; Petrosyan et al., 2014). In combination with an astrocytic selective promoter (Lee et al., 2008; Pfrieger and Slezak, 2012), these AAVs are expected to express the gene of interest selectively in astrocytes in specific brain regions.

The disadvantages of using viral vectors to express genes in astrocytes should not be overlooked. The most obvious concerns are tissue damage and reactive gliosis induced by viral injection. Reactive astrocytes display more robust, frequent and widely-spread intracellular Ca^2+^ activity, and intercellular coupling and Ca^2+^ waves are exaggerated among reactive astrocytes (Agulhon et al., 2012). A recent study showed that AAV 2/5 vector can be used to induce astrogliosis and disrupt the glutamate-glutamine cycle in astrocytes, which led to glutamate-reversible hyperactivity of nearby neurons (Ortinski et al., 2010). The primary methods for assessing whether or not astrocytes are pathologically transformed by viral infection are through morphological studies and GFAP expression levels (Xie et al., 2010; Shigetomi et al., 2013a). However, it is likely that more subtle undetected changes in astrocytes occur that could influence their functional interactions with surrounding cells.

Another challenge of using viral injection to express constructs in astrocytes is cell-specificity. AAVs show tropism to all cell types in the CNS (Aschauer et al., 2013; Gholizadeh et al., 2013; Petrosyan et al., 2014; Yang et al., 2014). Even with an astrocytic-specific promoter, it is important to carefully verify astrocyte specific transduction using the most sensitive methods available. A low level of GPCR expression in non-astrocyte cell types could lead to significant downstream signaling and confound the interpretation of findings.

One final consideration to keep in mind is that it is currently impossible to transduce functionally distinct populations of astrocytes in a manner analogous to transducing a subpopulation of functionally distinct neurons; this will only be solved as new subpopulation specific astrocyte transcriptional units are identified.

## Current knock-out models for selective inactivation of astrocytic signaling

### Specific GPCR knockout models

To date, most investigators have used optogenetic, pharmacogenetic or pharmacological tools to determine the consequences of activating astrocyte signaling cascades (Agulhon et al., 2008). None of these approaches recapitulate the complex regulation of signaling cascades occurring *in vivo*. Findings from these studies (see review by Agulhon et al., 2008) provide insight into the potential outcome(s) of astrocyte signaling. However, one has to keep in mind that these highly artificial types of stimulation may lead to outcomes that rarely, or perhaps never, occur in physiology. To determine the functional significance of astrocytic signaling, a more powerful approach is to demonstrate that the loss-of-function of a particular pathway affects physiological processes such as synaptic transmission or behavior. Here we review current knockout (KO) and conditional KO (cKO) models for deleting specific astrocytic signaling pathways (Table 2).

**Table 2:**
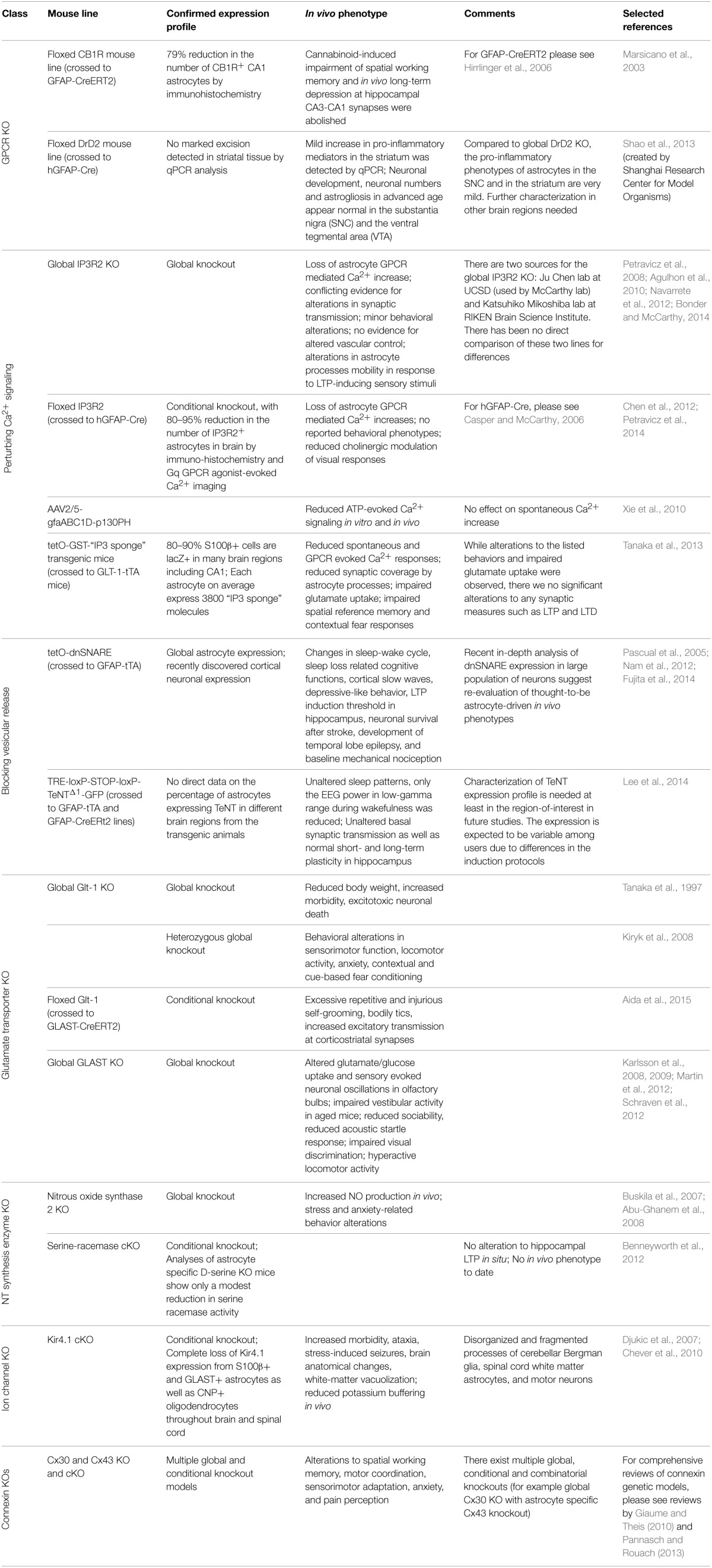
**Current molecular tools for perturbing astrocyte signaling *in vivo***.

#### GFAP-CB1R-KO

One of the first GPCRs targeted for a cKO in astrocytes was the cannabinoid type-1 receptor (CB1R), a GPCR predominately coupled to Gi signaling (Han et al., 2012). GFAP-CB1R cKO mouse line was accomplished by crossing floxed CB1R mouse line with an inducible Cre system driven by the GFAP promoter (GFAP-CreERT2) (Hirrlinger et al., 2006). Following tamoxifen administration, CB1R expression was reduced in GFAP^+^ astrocytes in the brain, providing the first model to study the contribution of astrocytes in cannabinoid induced working memory impairment *in vivo*. Han et al. found that conditional knockout of astrocytic CB1R abolished cannabinoid-induced impairment of spatial working memory and *in vivo* long-term depression at hippocampal CA3-CA1 synapses. In contrast, a cKO of CB1R in glutamatergic or GABAergic neurons did not rescue cannabinoid induced deficit (Han et al., 2012). This study revealed significant impact of a single astrocyte GPCR signaling system on synaptic modulation and behavior, as well as presented an elegant *in vivo* system for temporal control of astrocytic signaling.

#### GFAP-DRD2-cKO

In 2013, an astrocyte dopamine D2 receptor (DRD2) conditional knockout mouse was generated to study the role of astrocytic GPCR signaling in aging-related neuroinflammation (Shao et al., 2013). DRD2 couples to Gi (Missale et al., 1998) and is expressed in astrocytes *in vivo* (Khan et al., 2001). During aging, DRD2 expression is downregulated in the brain (Kaasinen et al., 2000), suggesting potential involvement of DRD2 in aging related neuroinflammation. In this study DRD2-deficient astrocytes were found to produce more proinflammatory mediators compared to wild-type astrocytes. Further, this effect appears to be mediated through a decrease in αB-crystallin (CRYAB) signaling, a small heat-shock protein known to negatively regulate pro-inflammatory mediator production and to display neuro-protective effect (Ousman et al., 2007). Interestingly, DRD2-deficient astrocytes also show robust GFAP upregulation, and a reactive morphology in the substantia nigra and the striatum of aged mice (Shao et al., 2013), suggesting the possible link between astrocytic GPCR signaling and age-related impairments in cognitive and motor function.

### Models for removing or partially removing astrocytic Ca^2+^ fluxes *in vivo*

#### IP3R2 germline and conditional IP3R2 knockout

Astrocytic Gq-GPCR/PLC/IP3 signaling is the most intensely studied pathway in the proposed modulation of neuronal activity and cerebral blood flow by astrocytes and has been the subject of numerous reviews (Haydon and Carmignoto, 2006; Agulhon et al., 2008, 2012; Fiacco et al., 2009; Halassa and Haydon, 2010; Hamilton and Attwell, 2010). The activation of this pathway results in the release of Ca^2+^ from IP3 receptor (IP3R) regulated intracellular stores in the endoplasmic reticulum. Astrocytes express only one of three subtypes of this receptor, IP3R type 2 (IP3R2) (Sharp et al., 1999; Holtzclaw et al., 2002; Foskett et al., 2007; Hertle and Yeckel, 2007). The first functional report to confirm this was published by Petravicz et al. (2008), in which the germline IP3R2 knockout mouse (generated by the Ju Chen lab; Li et al., 2005) was found to lack somatic increases in intracellular Ca^2+^ upon activation of multiple subtypes of Gq-GPCRs known to exist on astrocytes. Further experiments using this model have confirmed that astrocytes lack Gq-GCPR elicited, IP3R-dependent Ca^2+^ signals in their processes and soma (Agulhon et al., 2010; Di Castro et al., 2011; Panatier et al., 2011; Takata et al., 2011; Navarrete et al., 2012; Tamamushi et al., 2012; Nizar et al., 2013). The IP3R2 KO mouse is fertile, displays no overt alterations in brain development, and displays no obvious behavioral alterations (in contrast to the IP3R1 KO, the primary neuronal IP3R Matsumoto and Nagata, 1999). Due to these features, this mouse model has become one the most utilized mouse models in astrocyte research. Use of this model has led to novel findings (Panatier et al., 2011; Navarrete et al., 2012; Haustein et al., 2014; Perez-Alvarez et al., 2014), several of which have been contradictory with previously held theories concerning astrocyte-neuron communication and vascular control (Fiacco et al., 2007; Petravicz et al., 2008; Agulhon et al., 2010; Nizar et al., 2013; Takata et al., 2013; Bonder and McCarthy, 2014). Recently, evidence of residual Ca^2+^ signaling of a non-IP3R origin has been published using genetically encoded Ca^2+^ indicators (GECIs) in the IP3R2 KO model (Haustein et al., 2014; Kanemaru et al., 2014), further illustrating the model's usefulness for discovery of novel signaling events in astrocytes; importantly, there is no evidence suggesting that this residual Ca^2+^ signaling is regulated by neuronal activity.

Due to its restricted expression pattern in the CNS, a germline knockout of IP3R2 provides a clean and reliable model to block the release of intracellular Ca^2+^ in astrocytes elicited by Gq-GPCR activity *in vivo*. However, this model suffers in that it lacks tissue specificity, as do all germline knockout models. IP3R2 is expressed in multiple tissues outside the CNS including the heart (Li et al., 2005), pancreas (Orabi et al., 2012), lungs, liver, and kidneys (Fujino et al., 1995); this leads to potential confounding issues in the use of this model *in vivo* when assessing the role of astrocyte GPCR-dependent Ca^2+^ fluxes in behavior. Additionally, concerns over compensation due to the role of intracellular Ca^2+^ signaling during development have been raised regarding this model; however to date no evidence for altered development leading to compensation has been reported.

In an attempt to address some of these issues, a conditional IP3R2 knockout mouse model was developed by our laboratory. This model was generated by crossing the original floxed IP3R2 mouse developed by Dr. Ju Chen at UCSF (Li et al., 2005) to a GFAP-Cre recombinase mouse (Stehlik et al., 2006) to restrict the deletion of IP3R2 to GFAP^+^ cells in the CNS. This model recombines the floxed IP3R2 allele at a high rate (>80–85% GFAP^+^ cells lack IP3R2), significantly reducing the number of astrocytes responding to Gq-GPCR activation or neuronal activity in multiple brain regions (Petravicz et al., 2008; Chen et al., 2012). The use of the GFAP-Cre system spatially and temporally restricts the IP3R2 deletion to GFAP^+^ glia, thereby making it more appropriate for *in vivo* analysis such as behavioral characterization and reduces potential developmental compensation. Our lab recently published a behavioral analysis of the IP3R2 cKO mice and found no significant alteration to behavior (Petravicz et al., 2014). Most importantly, no alterations to learning and memory as assessed by the Morris Water Maze test were observed in these mice, despite previous literature proposing an important role for astrocyte IP3R-mediated Ca^2+^ signaling in hippocampal LTP. It is unlikely that developmental compensation occurs such that alternative ions substitute for Ca^2+^ in physiological processes or that global rewiring of neuronal circuits occurs to compensate for the loss of astrocyte Ca^2+^ fluxes. Nevertheless, to completely rule out developmental compensation, it will be necessary to prepare inducible IP3R2 cKO mice using mice expressing floxed IP3R2 and an astrocyte specific inducible Cre system.

#### IP3 sponges

Activation of Phospholipase C beta (PLCβ) and sequential release of IP3 are the key steps in Gq-GPCR mediated intracellular Ca^2+^ elevations. Traditional approaches to abolish Gq-GPCR mediated Ca^2+^ elevation in astrocytes include chelating intracellular Ca^2+^ with BAPTA, or preventing IP3-mediated release of Ca^2+^ from ER using IP3R2 KO mice. Recently, the Pleckstrin Homology domain of PLC-like protein (p130PH), which binds cytosolic IP3 molecules, was used to suppress astrocytic Ca^2+^ signaling *in vitro* and ATP-induced astrocytic Ca^2+^ responses *in vivo* (Xie et al., 2010). In this study, p130PH was selectively expressed in cortical astrocytes *in vivo* using rAAV2/5 vector in combination with a specific astrocyte promoter, gfaABC(1)D (Lee et al., 2008; Xie et al., 2010). p130PH transduced astrocytes in the somatosensory cortex exhibited reduced amplitude and frequency of Ca^2+^ activity in response to direct ATP application on cortex compared to non-transduced astrocytes, whereas the characteristics of spontaneous Ca^2+^ activity in p130PH-transduced astrocytes remained unchanged (Xie et al., 2010). Therefore, p130PH serves as a more selective tool to suppress Gq-GPCR induced Ca^2+^ elevations without chelating Ca^2+^ activity in astrocytes completely. This system serves as a nice addition to IP3R2KO and IP3R2 cKO mouse lines to distinguish astrocyte functions regulated by ER released Ca^2+^ vs. channel mediated Ca^2+^ *in vivo*.

The expression of a fragment of the IP3 binding domain of IP3R1, an “IP3 sponge” (Iwasaki et al., 2002) can also be used to suppress IP3 induced Ca^2+^ release in astrocytes *in vivo* (Tanaka et al., 2013). These investigators found that suppression of astrocyte Ca^2+^ responses affected several behavioral responses, but no underlying evidence for alterations to synaptic transmission was found. Surprisingly, astrocytes expressing the “IP3 sponge” exhibited process retraction surrounding synapses, which was attributed to underlie the behavioral phenotypes. Recently, evidence that astrocyte processes retract in response to LTP-inducing stimuli *in vivo* was reported, and that this feature was lacking in IP3R2 KO mice (Perez-Alvarez et al., 2014). These findings appear to contradict those reported by Tanaka et al. (2013). Further comparison between these two methods of blocking astrocyte Ca^2+^ increases will be required to clarify this contradiction.

### Blocking vesicular release from astrocytes with dnSNARE or tetanus toxin

Ca^2+^ dependent release of neurotransmitters from astrocytes, termed “gliotransmission,” is one of the most important concepts presented in glial biology over the past several decades (Araque et al., 2014). While several mechanisms have been suggested to underlie gliotransmission, most studies support a process dependent on a vesicular release system (i.e., a SNARE dependent process) similar to that found in neurons (Zorec et al., 2012; Sahlender et al., 2014). To test the significance of SNARE-mediated gliotransmission to synaptic function, a mouse line that expresses a dominant negative form of SNARE (dnSNARE) in astrocytes was developed in our laboratory in 2005 (Pascual et al., 2005). This line was prepared by coinjecting three independent constructs (tetO-lacZ, tetO-dnSNARE, and tetO-eGFP) into fertilized zygotes. In dnSNARE transgenic mice, the expression of the cytosolic portion of the SNARE domain of synaptobrevin 2, lacZ, and eGFP are controlled by tetracycline regulatory system. When crossed with GFAP-tTA mice, the expression of dnSNARE, lacZ, and eGFP are independently controlled by doxycycline. In the absence of doxycycline, dnSNARE is expressed and interferes with SNARE-dependent vesicular release. A limitation of this model is that since dnSNARE was not directly tagged, there is no way *in situ* to verify that it is not expressed in cells other than astrocytes. Nevertheless, the dnSNARE mice have been used in a large number of studies to demonstrate a role for gliotransmission in synaptic transmission, synaptic plasticity, as well as behavior. However, a recent paper performed an in-depth analysis of dnSNARE mice and found that that the expression of dnSNARE was also expressed by a large population of neurons (Fujita et al., 2014). Given the critical role of the SNARE complex in neurotransmitter release, these findings bring into question the validity of this model and the findings obtained using this system.

Studies using dnSNARE mice suggest that SNARE-mediated astrocytic release of ATP and subsequent adenosine receptor activation regulates neuronal excitability and synaptic plasticity in many brain regions as well as modulate certain behaviors including sleep (Nam et al., 2012). The dnSNARE mice exhibit a weak sleep phenotype under basal conditions, as well as an attenuated “rebound” response to sleep deprivation (Halassa et al., 2009). Cortical slow oscillations, a rhythm characterizing non-rapid eye movement (non-REM) sleep was also found impaired in dnSNARE mice (Fellin et al., 2009). Further, the hippocampal dependent memory deficits produced by sleep deprivation were rescued in dnSNARE mice (Florian et al., 2011). These studies suggest that astrocytes release ATP in vesicular manner and that this plays an important role in sleep patterns and cortical oscillations.

Tenanus toxins (TeNTs) are known to interfere with synaptic vesicular release as well as other processes dependent on vesicular protein trafficking (Galli et al., 1994). Recently, a transgenic model system was developed using TeNT to block vesicular release in astrocytes *in vivo* (Lee et al., 2014). This transgenic model took advantage of both the tetracycline inducible regulatory system and the Cre-dependent inducible system to block vesicular release from astrocytes. A transgenic line was prepared that contained the tetracycline response element (TRE) followed by a floxed stop cassette and a cassette that when expressed led to the expression of eGFP tagged TeNT (TRE-loxP-STOP-loxP-TeNT^Δ1^-GFP). In this system, the expression of TeNT required Cre expression to remove the floxed stop cassette and tetracycline transactivator (tTA) to activate the TRE. TRE-loxP-STOP-loxP-TeNT^Δ1^-GFP mice were crossed with GFAP-tTA and GFAP-CreERt2 lines to create a triple transgenic mouse line. In the triple transgenic mice TeNT can be expressed in astrocytes following tamoxifen treatment; the expression of TeNT is suppressed in the presence of doxycycline. In this model, astrocyte expression of TeNT requires that two different transgenes driven by the GFAP promoter be expressed in the same astrocyte markedly increasing the probability of astrocyte specific expression. Further, as TeNT was directly tagged with eGFP, it is possible to identify all cells expressing TeNT. In contrast to the impaired sleep pattern in dnSNARE mice, astrocytic TeNT-expressing mice showed unaltered sleep patterns compared to triple transgenic mice without tamoxifen or double transgenic mice with tamoxifin (Lee et al., 2014). Studies using this mouse line indicate that basal synaptic transmission as well as normal short- and long-term plasticity in hippocampus *in situ* is not altered by the expression of TeNT (Lee et al., 2014); these findings bring into question the concept that astrocytes release gliotransmitters that modulate synaptic transmission and plasticity via a vesicular dependent process. Interestingly, the EEG power in low-gamma range during wakefulness was reduced, whereas the EEG power during but not non-REM sleep remain unchanged (Lee et al., 2014). These observations in sleep regulation from astrocytic TeNT-expressing mice do not match with those from dnSNARE mice.

Memory deficit was also detected in the mice expressing TeNT in astrocytes using novel object recognition test (Lee et al., 2014). Fast local field potential oscillations in the gamma frequency are closely correlated with many cognitive functions, including learning, memory storage and retrieval and attention (Basar-Eroglu et al., 1996). Astrocytic TeNT-expressing mice did not show deficit in other behavior tests that involves simpler form of memory processing or are less dependent on cortical processing. The reduction of gamma oscillation power and significant deficit in novel object recognition suggest that the fast neural circuit oscillations are regulated by astrocytes (Lee et al., 2014).

### Mouse models for assessing astrocyte neurotransmitter regulation

#### Glutamate transporter knockout mouse models

Astrocytes are responsible for 80–90% of glutamate reuptake in the brain (Tzingounis and Wadiche, 2007), and two of the five glutamate transporters (GluTs) are primarily expressed in astrocytes: Glt-1/EAAT2 and GLAST/EAAT1 (Danbolt, 2001; Huang and Bergles, 2004). There are currently germline knockout mouse models for both Glt-1 (Tanaka et al., 1997) and GLAST (Harada et al., 1998); however their use *in vivo* has been limited. The Glt-1 germline knockout mouse model suffers from reduced body weight, increased morbidity, and progressive neuronal death due to excitotoxocity (Tanaka et al., 1997). This limits the ability to conduct *in vivo* experiments, which typically require older mice, to examine the role of astrocytic glutamate reuptake via Glt-1. Recently, the heterozygous Glt-1 (Glt-1 Het) knockout model has become an attractive model for studying the role of Glt-1 *in vivo*. The Glt-1 Het model does not suffer from the more obvious adverse effects of the full Glt-1 knockout, and displays several interesting behavioral phenotypes and has allowed for the study of Glt-1 in several brain pathologies (Kiryk et al., 2008). The GLAST knockout mice are viable, enabling *in vivo* studies. GLAST is primarily expressed in the cerebellum and olfactory bulb (Regan et al., 2007), and this mouse model has led to interesting findings concerning the physiological (Martin et al., 2012; Schraven et al., 2012) and pathological (Karlsson et al., 2008, 2009) functions of GLAST. Recently, a Glt-1 floxed mouse model has been developed (Aida et al., 2015). Induced knockout of Glt-1 in adult animals resulted in development of repetitive behaviors and alterations to excitatory transmission due to reduced glutamate uptake. The floxed Glt-1 model when combined with inducible Cre systems will open up new avenues of research into the role of Glt-1 *in vivo* that were not possible due to the lethality of the germline Glt-1 KO.

#### Nitrous oxide synthase 2 knockout

In the central nervous system nitric oxide serves a number of roles, and has been shown to act at glutamatergic synapses to enhance glutamate release (Garthwaite, 2008). While neurons in many brain regions are known to produce and release NO via nitric oxide synthase (nNOS), this does not account fully for the activity of NO in several brain regions where excitatory neurons lack nNOS expression. Astrocytes are known to express all three isoforms of NOS, and are the sole expressers of an inducible form of NOS (iNOS or NOS2) that is activated in response to physiological stress in a Ca^2+^ dependent manner (Murphy, 2000; Buskila et al., 2005; Amitai, 2010). Astrocyte-derived NO has been shown to enhance LTP of presynaptic afferents in the spinal cord, as well as enhance synaptic transmission in the neocortex in acute slice preparations (Ikeda and Murase, 2004; Buskila and Amitai, 2010). *In vivo* evidence for astrocytic-derived NO being a modulator of neuronal transmission has primarily come from the use of a NOS2 knockout mouse, in which it was discovered that deletion of the calmodulin-binding domain of NOS2 led to a net increase in overall NO concentrations in the brains of mutant mice. Interesting, the increase in NO originated from astrocytes through an alternate mechanism without alterations in the relative levels of NOS isoforms (Buskila et al., 2007). Further, these mice display stress and anxiety-related alterations to behavior suggesting a role for astrocyte-derived NO in the modulation of neural circuits (Abu-Ghanem et al., 2008). Currently, the role of astrocyte-derived NO in modulation of neuronal circuit activity remains an understudied area of glial research.

#### Astrocytic serine-racemase conditional knockout

D-serine has long been considered one of the three primary gliotransmitters along with ATP/adenosine and glutamate, with putative astrocyte derived D-serine reported to modulate neuronal NMDA receptors (Panatier et al., 2006; Billard, 2008; Oliet and Mothet, 2009; Martineau, 2013; Shigetomi et al., 2013b; Sild and Van Horn, 2013). The enzyme responsible for D-serine production (serine racemase, SR) was initially found to be primarily expressed by astrocytes with some modest expression in neurons (Schell et al., 1995; Mothet et al., 2005). However, more recent studies have called this expression pattern into question (Miya et al., 2008; Ding et al., 2011; Ehmsen et al., 2013). The most recent study of SR localization in mice and human brains finds that nearly all immunostaining for SR is found in neurons and not astrocytes (Balu et al., 2014). Recently, cell type specific knockouts of SR were generated to examine the relative contributions of astrocytes and neurons in the forebrain of mice (Benneyworth et al., 2012). The astrocyte specific knockout of SR led to a modest (~15%) reduction in SR expression, while the neuronal knockout in forebrain neurons reduced SR by much larger amounts (~65%). Further, the neuronal specific SR knockout displayed alterations to LTP at hippocampal synapses that were not found in the astrocyte SR knockout. These findings raise new questions in how astrocytes may be regulating D-serine availability in the brain, which the astrocyte specific SR knockout will be crucial to resolving.

### Knockout models for astrocyte membrane channels

#### Kir4.1

One of the major roles in the CNS for astrocytes is the buffering of potassium ions in response to neuronal activity. Astrocytes express a variety of potassium channels, but among them Kir4.1 plays a predominant role in their K^+^ buffering capacity (Takumi et al., 1995; Higashi et al., 2001; Djukic et al., 2007). A germline full Kir4.1 knockout model has provided insight into the role of potassium buffering in response to hyperammonemic conditions (Stephan et al., 2012) and the channel's role in regulating astrocyte membrane potential during development (Seifert et al., 2009). An astrocyte conditional knockout model for Kir4.1 was generated by the McCarthy lab to provide a cleaner animal model alternative to the germline KO (Djukic et al., 2007). The conditional knockout astrocytes display reduced glutamate clearance and decreased resting membrane voltage, while neuronal plasticity was enhanced implicating Kir4.1 as an important mediator of extracellular potassium regulation. These finding have been confirmed in an *in vivo* study utilizing the Kir4.1 cKO, with the cKO mice found to have reduced capacity to regulate extracellular potassium levels compared to controls (Chever et al., 2010). Usage of this model in awake mice to examine the effect of altered potassium homeostasis may provide unique insights into how astrocytes regulate neuronal networks.

#### Connexins

Astrocytes in the brain exist not only as single units occupying a discrete domain, but also as a network of cells connected by connexin (Cx) gap junctions. Astrocytes express two major connexin proteins (Cx43 and Cx30) with germline and conditional knockout mouse models existing (Dermietzel et al., 2000; Teubner et al., 2003; Wiencken-Barger et al., 2007). These mouse models display a variety of alterations to behavior, synaptic transmission, metabolic support, and ion homeostasis (Giaume and Theis, 2010; Pannasch and Rouach, 2013). However, there are a large number of open questions concerning the role of gap junction communication and astrocyte networks *in vivo*. Currently, the only *in vivo* experiments in these models have involved behavioral studies, indicating a significant impact on neuronal circuit function (for an excellent review please see Pannasch and Rouach, 2013). Further exploration of neuronal activity *in vivo* utilizing these models, as well as generation of inducible knockout systems, represents novel avenues of research for understanding astrocyte network function.

### Current limitations in genetically targeting astrocytes

At this time, there is not a single gene delivery system that can be used to exclusively express transgenes or recombine naïve genes in astrocytes. The common astrocyte marker proteins (GFAP, S100b, glutamine synthetase, aquaporin 4, connexin43, GLAST, GLT1, ALDH1L1) are either expressed in alternate subsets of mature CNS cells (Dunham et al., 1992; Zhuo et al., 2001; Su et al., 2004; Hachem et al., 2005; Regan et al., 2007; Donato et al., 2013), in progenitor CNS cells that give rise to multiple CNS cell types (Hartfuss et al., 2001; Casper and McCarthy, 2006), or are expressed outside the CNS (Jessen et al., 1990; Rinholm et al., 2007; Darlot et al., 2008; Meabon et al., 2012; Rutkovskiy et al., 2012; Jesus et al., 2014; Kato et al., 2014). The GFAP transcriptional regulatory unit (TRU) is probably the best characterized and most frequently used TRU for regulating gene expression in astrocytes and serves as a good example to illustrate the difficulties in targeting genes to astrocytes. The GFAP TRU is active in progenitor cells that give rise to astrocytes, neurons, and oligodendrocytes (Casper and McCarthy, 2006). Even in the mature CNS, certain populations of neurons express GFAP (Zhuo et al., 2001; Su et al., 2004; Regan et al., 2007). Further, in the periphery, non-myelinating peripheral glia (Jessen et al., 1990) as well as certain populations of non-neural cells (e.g., stellate cells in the liver) are GFAP^+^ (Lim et al., 2008) and will express transgenes driven by the GFAP TRU. To avoid transgene expression in progenitor cells, many laboratories have developed inducible gene regulatory systems (Casper and McCarthy, 2006; Hirrlinger et al., 2006; Mori et al., 2006). This approach circumvents gene expression in progenitors during development but does not affect the expression of transgenes in peripheral glia, adult stem cells or small populations of neurons that normally express GFAP. How much of a problem this presents depends on the question being asked. When studying a transgene in the mature CNS that can be assumed not to affect developmental processes nor lead to a peripheral phenotype it is reasonable to use many of the available astrocyte TRU to drive transgenes to astrocytes. For example, the GFAP TRU can be used to express eGFP, GCaMP, or DREADD receptors that must be activated by an exogenous ligand without preventative concern about developmental expression. However, when using the GFAP TRU to drive bioactive molecules such as DREADD receptors or inducible Cre recombinase, it is important to remain cognizant that in addition to astrocytes, peripheral GFAP^+^ cells, adult stem cells, and certain neurons will also be affected. Alternatively, when expressing molecules that innately affect biological processes (e.g., a dominant negative mutation, constitutively active signaling molecule or Cre recombinase), it is very important to consider the consequences of expression during development.

The above discussion assumes that the astrocyte TRU is acting with the fidelity of the endogenous TRU. Unfortunately, this is often not the case and is largely dependent on the genomic construct used to prepare the TRU. Most typically, investigators use a fragment of the TRU to drive transgene expression. As transgenes generally integrate somewhat randomly at active sites in the genome, the activity of surrounding genomic regulatory units can markedly affect transgene expression levels as well as the cells the transgene is expressed. This problem is markedly reduced using a BAC approach where very large genomic segments containing the TRU and inserted transgene are used to prepare transgenic lines. One final difficulty is that there are no astrocyte TRU systems that can be used to target subpopulations of astrocytes. Currently, the only way to genetically-manipulate subpopulations of astrocytes is to transduce these cells using viral vectors. Unfortunately, this generally restricts the size of the TRU used to target astrocytes and requires viral injection that may lead to subtle or striking changes in astrocyte function.

In summary, genetic manipulation of astrocytes is a very powerful tool for assessing the role of these cells in physiology, disease, and behavior. However, just as one has to verify the specificity of pharmacological reagents, great care must be used to insure the specificity of genetic manipulations.

## Emerging technologies for manipulating astrocytic signaling *in vivo*

### Temporal control of GPCR signaling using optogenetic GPCRs

Optogenetically activated GPCR signaling is a reasonable alternative to ChR2 stimulation in astrocytes. There are two systems based on adrenergic receptors: Opto-alpha-1 (Gq linked) and opto-beta-1 (Gs linked) (Airan et al., 2009). Activation of these systems use similar experimental methods as optogenetics, but have the advantage of activating endogenous signaling cascades that exist in astrocytes. To date, these systems have not been tested for astrocytes *in vivo*, only in astroglia in culture (Figueiredo et al., 2014). While the Gq-linked opto-alpha-1 predictably elicited Ca^2+^ increases via release from intracellular stores, the Gs-linked opto-beta-1 was found to also trigger Ca^2+^ increases in a cyclic AMP-dependent manner. In culture systems, it has been shown that the Gβγ subunit of Gs-coupled GPCRs is capable of directly gating IP3Rs (Zeng et al., 2003). Recently, experiments in HEK293 cells discovered that IP3R2 complexes with Gαs and type 6 adenyl cyclase (AC6), and facilitates crosstalk between the two signaling pathways (Tovey et al., 2008, 2010). Further study into the mechanism behind increases in Ca^2+^ in astrocytes may lead to the identification of novel pathways regulating Ca^2+^ signaling.

Recently, the concept of using opsin based-pigments to develop optogenetic tools for modulating GPCR signaling was suggested (Koyanagi and Terakita, 2014). One of the candidates is melanopsin (OPN4), a Gq-coupled opsin that is originally found in a subtype of retinal ganglion cells (Hatori and Panda, 2010; Sexton et al., 2012). Several groups have used ectopic expression of OPN4 to control intracellular Ca^2+^ dynamics in neurons (Koizumi et al., 2013). In 2013, Karunarathne and colleagues used non-rhodopsin opsins to activate native Gq, Gi/o, and Gs signaling in localized regions of a single cell, and were able to gain spatial-temporal control over immune cell migration (Karunarathne et al., 2013b) as well as neurite initiation and extension (Karunarathne et al., 2013a). These studies suggest high potential of opsins as optogenetic GPCRs for *in vivo* astrocyte research.

### Studying G-protein independent signaling using biased DREADDs

One important aspect of GPCR signaling in astrocytes that has rarely been explored is the role of G-protein independent signaling. Endogenous GPCR activation not only initiates signaling via heterotrimeric G proteins, but also recruits proteins of the arrestin family, which act as scaffolding proteins and promote G protein-independent signaling (Pierce et al., 2002; Rajagopal et al., 2010; Shukla et al., 2011). Research has shown that arrestin 3 (β-arrestin 2) is expressed in astrocytes *ex vivo* (Bruchas et al., 2006; McLennan et al., 2008), and it is involved in kappa opioid receptor (KOR)-induced proliferation (McLennan et al., 2008; Miyatake et al., 2009), reduction of chemical-induced apoptosis (Zhu and Reiser, 2014), CXCR7 mediated inflammatory response (Odemis et al., 2012; Lipfert et al., 2013) and beta 2-adrenergic receptor (β2AR)-mediated glycogenolysis (Dong et al., 2001; Du et al., 2010) in astrocytes. As a scaffolding protein, β-arrestins also mediate internalization and ubiquitylation for many ion channels and transporters expressed in astrocytes (Shukla et al., 2011). As the list of signaling pathways that β-arrestins can regulate in astrocytes grows, it is important to dissect the relative contribution of G-protein independent signaling pathway to known functions of astrocytic GPCR signaling.

Recently, a modified Gq-DREADD that has strong biases toward arrestin-signaling was developed (Nakajima and Wess, 2012). This receptor was generated by introducing a point mutation within the highly conserved DRY motif [Rq(R165L)] of Gq-DREADD, which results in lack of ability to activate heterotrimetic G proteins. Therefore, CNO-induced Rq(R165L) activation has no effect on the levels of conventional second messages, but can promote CNO-dependent and arrestin-dependent signaling in biological systems (Nakajima and Wess, 2012). This novel GPCR represents an excellent tool to study the relative contribution of G protein-dependent and independent pathways in the known function of astrocytes, as well as reveal the physiological roles of astrocytic-arrestin signaling *in vivo*.

### Conditional Gαq/Gα11 KO

GPCR KO mouse lines for all the known Gα subunits have been developed to analyze the physiological function of GPCR signaling *in vivo* (Offermanns, 1999), many of which show deficiencies in CNS-related physiology (Offermanns, 2001). Although these KO mouse lines are not astrocyte specific, inducible and conditional Gαq/Gα11 KO mice are available (Wettschureck et al., 2001) and can be combined with astrocytic specific Cre mouse line to selectively knock out Gq-GPCR signaling in astrocytes.

The conditional Gαq/Gα11 KO system was developed to study the role of Gαq/Gα11 signaling in specific tissues without developmental problems exhibited in the constitutive KO mouse line for both genes. This mouse line was developed using the Cre/LoxP system where the Gαq gene (*gnaq*) is conditionally inactivated in Gα11 KO (*gna11*–/–) mice, which do not have obvious behavior defect (Stanislaus et al., 1998). Therefore, mice homozygous for *gnaq*^flox^ gene appear normal until Cre recombinase is introduced. The conditional Gαq/Gα11 KO mouse was first used with *MLC2a*-Cre to obtain cardiomyocyte-specific Gαq/Gα11 deficiency, resulting in a nearly complete recombination of *gnaq*^flox^ in cardiomyocytes (Wettschureck et al., 2001).

In 2006, the Gαq/Gα11 cKO mice was crossed to mice that express Cre under the control of the promoter of the Ca^2+^/calmodulin-dependent protein kinase IIα gene (*Camkcre4* mice) to generate forebrain specific and neuronal Gαq/Gα11 double KO mice (Wettschureck et al., 2004, 2006; Broicher et al., 2008), resulting in impaired endocannabinoid levels, increased seizure susceptibility (Wettschureck et al., 2006), and lack of maternal behavior in females (Wettschureck et al., 2004). The Gαq/Gα11 cKO line was also used to disrupt glial Gq/11 signaling in combination with Nestin-Cre mouse (Wettschureck et al., 2005), resulting in loss of Gq/11 in the neural stem cells that gives rise to both neurons and astrocytes. Although the Gq/11 signaling deficiency did not cause gross morphological changes in the developing nervous system, pups with insufficient Gq/11 signaling suffers from hypothalamic growth hormone deficiency and somatotroph hypoplasia, dwarfism, and anorexia (Wettschureck et al., 2005). Given the availability of astrocytic-specific Cre mice, it will be possible to isolate the contribution of astrocytic Gq/11 signaling *in vivo*.

## Summary and future directions

There is no doubt that genetic tools will play an important role in understanding the role of astrocytes in physiology, behavior, and neurological disorders. Studies cited above provide strong evidence that astrocytes are doing much more than simply insulating synapses and providing nutrients to neurons. It is not surprising that findings using genetic tools may conflict with previous findings using pharmacological methods to perturb astrocyte function. It is also not surprising that different genetic approaches (e.g., dnSNARE and tetanus toxin expression) may yield different results. These conflicts require additional studies using multiple tools (pharmacological and genetic) to refine our understanding of astrocyte function. Currently, there are several important limitations in the genetic tools available for perturbing astrocyte function *in vivo*. First, we are extremely limited in the transcriptional units (promoters) available for expressing molecules in astrocytes. Currently, all available transcriptional units drive transgene expression in cells other than astrocytes. Further, during development, most astrocyte promoters drive gene expression in progenitor cells that give rise to neurons, oligodendrocytes, and astrocytes (Casper and McCarthy, 2006). Consequently, it is necessary to use inducible systems linked to astrocyte promoters to avoid transgene expression in progenitors during development. Second, when expressing transgenes that drive function, it is important to remember that the transgene may be overexpressed and targeted to cellular compartments not normally found. While this can be partially overcome using an inducible gene regulatory system, it is likely that this will remain a caveat until we know a great deal more about the cell machinery in astrocytes that target molecules to specific cellular compartments. Third, we currently lack promoters that can be used to drive transgene expression in subtypes of astrocytes. Subtype specific astrocyte promoters will enable important advances with respect to the heterogeneity of astrocytes as well as their different functional roles.

In summary, while genetic manipulation of astrocytes is in its early stages of development, genetic models have already provided important insight into the role of astrocytes in physiology, behavior, and neurological diseases. It seems very likely that future advances in this field will depend largely on genetic approaches currently available as well as those under development.

### Conflict of interest statement

The authors declare that the research was conducted in the absence of any commercial or financial relationships that could be construed as a potential conflict of interest.
